# Conquering aging-related immunosenescence and tumor immune escape

**DOI:** 10.3389/fimmu.2026.1785351

**Published:** 2026-03-10

**Authors:** Shanshan Zhang, Bo Yang, Mingyi Xu, Ying Bing, Jiwang Zhang, Yuanxin Wang, Chaohui Zheng, Gang Zhang, Leisheng Zhang

**Affiliations:** 1 Shandong Provincial Key Medical and Health Laboratory of Blood Ecology and Biointelligence, Science and Technology Innovation Center, The Fourth People’s Hospital of Jinan Affiliated to Shandong Second Medical University, Jinan, China; 2 Jinan Key Laboratory of Medical Cell Bioengineering, Science and Technology Innovation Center, The Fourth People’s Hospital of Jinan Affiliated to Shandong Second Medical University, Jinan, China; 3 Department of Rheumatology, The Fourth Hospital of Jinan Affiliated to Shandong Second Medical University, Jinan, China; 4 College of Life Science and Technology & College of Basic Medicine, Shandong Second Medical University, Weifang, China; 5 Department of Otolaryngology, The Second Affiliated Hospital of Fujian Medical University, Quanzhou, China; 6 Department of General Surgery, The Fourth People’s Hospital of Jinan Affiliated to Shandong Second Medical University, Jinan, China

**Keywords:** aging, immunosenescence, SASP, tumor immune escape, tumor microenvironment (TME)

## Abstract

With global population aging, senescence has emerged as a key driver of tumorigenesis. Aging-associated molecular changes, including DNA damage, telomere shortening, and epigenetic dysregulation, increase malignancy, while immunosenescence and the senescence-associated secretory phenotype (SASP), reshape the tumor microenvironment to favor immune suppression and tumor escape. Aging also impairs antigen presentation, disrupts ligand-receptor signaling, and compromises tumor suppressive pathways. In the era of immunotherapy, elderly patients face reduced efficacy and increased resistance due to age-related immune remodeling. This review summarizes mechanisms of tumor immune escape in aging and discusses strategies to improve outcomes, such as senescent cell clearance, SASP modulation, immune potentiation, and combination therapies.

## 1 Introduction

As the global population continues to age, aging has been recognized as a major factor contributing to rising cancer incidence and mortality rates (1). Epidemiological studies as early as the late 19th to mid-20th century consistently showed that cancer incidence increases exponentially with age (2, 3). Aging is a complex biological process involving the gradual accumulation of damage and a decline in cellular and molecular functions. It affects not only individual cells through processes such as cellular senescence (4), but also disrupts tissue homeostasis and reduces overall physiological capacity (5).

Immunosenescence is a hallmark of aging and plays a central role in the increased susceptibility of older adults to infections, reduced vaccine responses, and the high prevalence of chronic diseases (6, 7). It is crucial to note that immunosenescence is not merely characterized by a simple decline in the number or function of immune cells. Rather, it represents a systematic process involving the structural degeneration of immune organs, remodeling of immune cell lineages, and dysregulation of immune signaling networks. Its impact extends across multiple tissues and organs, thereby profoundly altering the body’s capacity for immune surveillance against abnormal cells, including tumor cells and senescent cells (SnCs) (8–10).

It is important to recognize that aging promotes tumor progression through multiple mechanisms. The aging process is characterized by the accumulation of DNA damage (11, 12), telomere shortening (13, 14), and epigenetic alterations (15), all of which increase the tendency of malignant transformation (16). Although cellular senescence acts as an early antitumor barrier that facilitates the removal of damaged cells, the persistence of SnCs leads to the release of various factors through the senescence-associated secretory phenotype (SASP) (16, 17). SASP generates a microenvironment enriched with inflammatory mediators, growth factors, pro-angiogenic and pro-metastatic signals that also suppress immune activity (18, 19). This environment not only promotes malignant transformation in neighboring cells but also accelerates the progression of existing tumors.

**Figure 1:**
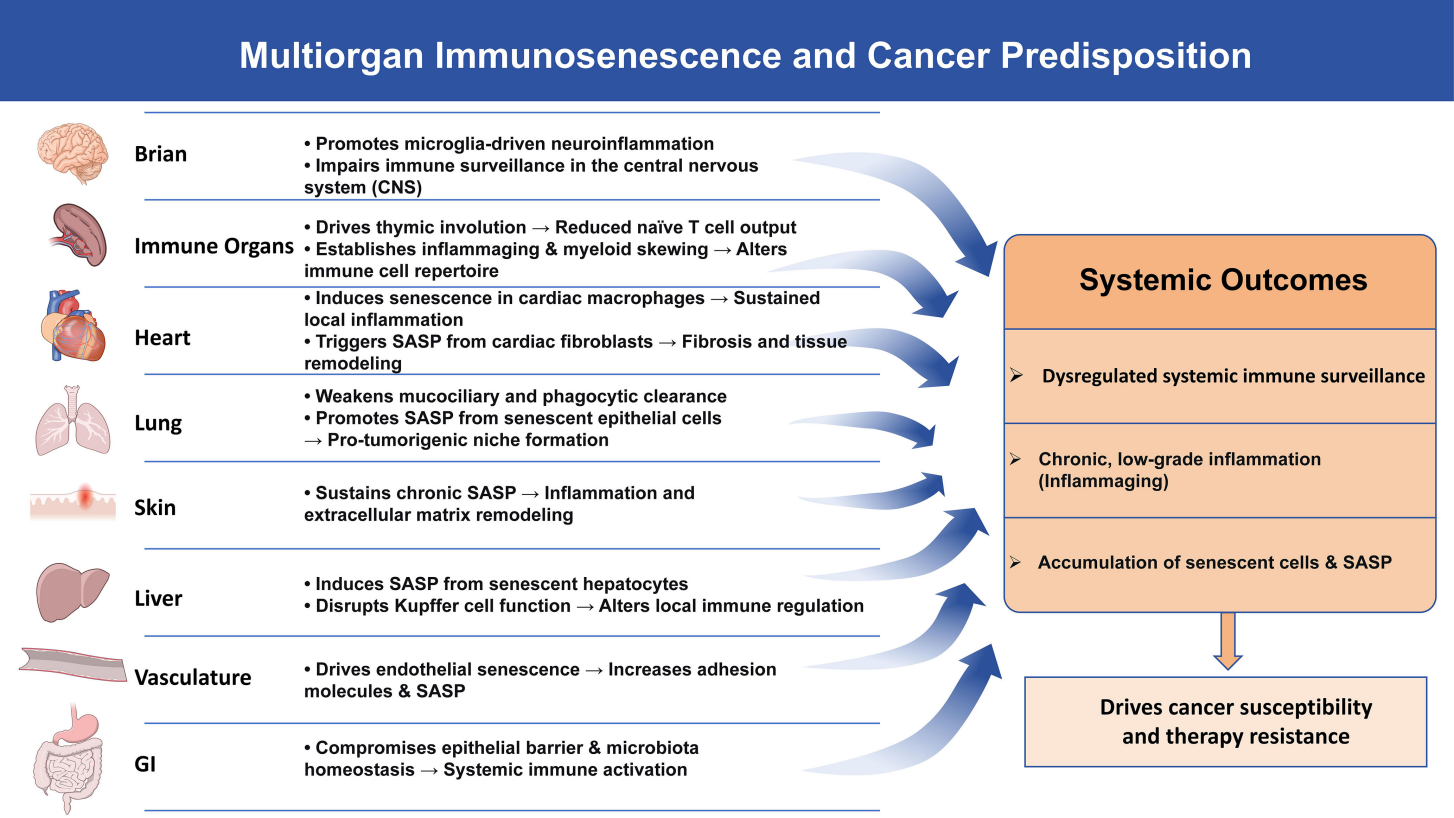
Multiorgan immunosenescence drives a pro-tumorigenic state. Immunosenescence actively disrupts tissue homeostasis across organs, driving neuroinflammation, SASP-mediated remodeling, and barrier dysfunction. These alterations converge to cripple systemic immune surveillance, sustain chronic inflammation, and foster SnCs accumulation, collectively establishing a milieu that heightens cancer susceptibility and undermines therapy efficacy. The image materials are provided by NIAID Visual & Medical Arts. SASP, senescence-associated secretory phenotype; SnCs, senescent cells.

In recent years, with the advancement of multi-omics and single-cell technologies, studies have further revealed that aging-associated immune and inflammatory alterations are pervasive across various tissues and organs, including the brain, lungs, liver, skin, and the vascular system. These findings indicate that both immunosenescence and the accumulation of SnCs exhibit significant tissue-specific patterns (20–23). Through mechanisms such as local immune cell dysfunction, amplification of chronic inflammation, and the formation of immunosuppressive microenvironments, they collectively drive systemic aging and create favorable conditions for tumorigenesis (**Figure 1**).

**Figure 2:**
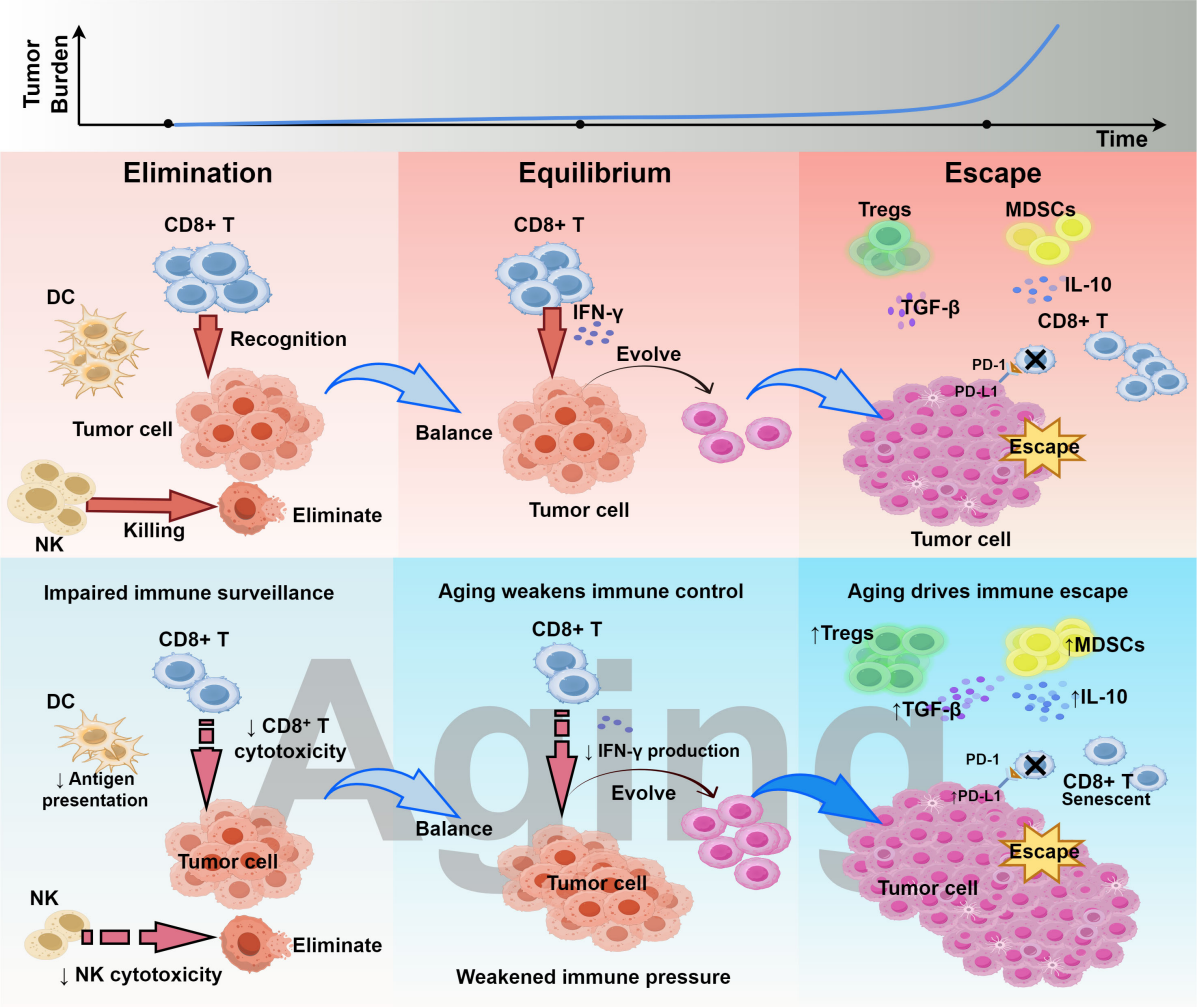
Aging reshapes cancer immunoediting. This figure illustrates the impact of immunosenescence on the three phases of cancer immunoediting: elimination, equilibrium, and escape. Aging weakens immune control during the elimination phase, creates a fragile balance in the equilibrium phase, and accelerates immune escape in the escape phase. This figure was drawn using Figdraw.

In the early 2000s, Robert Schreiber et al. proposed the concept of cancer immunoediting, which describes the interaction between the immune system and tumors through three dynamic phases: elimination, equilibrium, and escape. This concept demonstrated that the immune system can continuously recognize and eliminate emerging malignant cells, thereby protecting the host from cancer (24). However, in aging, key immune cell populations, especially natural killer (NK) cells, cytotoxic T cells, and macrophages, show reduced ability to detect and clear SnCs (25–28). As a result, SnCs accumulate in tissues, further enhancing SASP activity, thereby promoting chronic inflammation and tumor progression (29, 30). Thus, in the context of aging, the tumor immunoediting process is more likely to progress toward the “escape” phase. On the one hand, immunosenescence impairs the clearance capacity of effector immune cells; on the other hand, SnCs and their associated SASP continuously remodel the local immune microenvironment, providing tumor cells with a survival niche characterized by immune tolerance or even immunosuppression (**Figure 2**).

Over the past decade, cancer immunotherapy has driven significant advancements in oncology, such as immune checkpoint inhibitors (ICIs) (31, 32) and CAR-T cell therapy (33). These treatments enhance or restore the patient’s immune response to eliminate tumor cells and have significantly improved outcomes across multiple cancer types (34–39). However, in older patients, aging-related changes in the immune microenvironment create unique pathways that tumors can exploit to evade immune control (40). These alterations may weaken the antitumor effects of immunotherapy and increase the risk of adverse events, resulting in reduced efficacy and greater challenges in the elderly (41).

Given the global trend of aging and the rising proportion of cancer in the elderly, aging should not be regarded merely as a demographic variable in immunotherapy, but rather as a key biological process that profoundly reshapes the immune system and promotes tumor immune escape. Immunosenescence, accumulation of SnCs, and the SASP act together to establish an immunosuppressive microenvironment, thereby reducing the efficacy of ICIs and other immunotherapies in older patients. This review outlines the hallmarks of immunosenescence, synthesizes recent advances in the interplay between SnCs and the immune system, discusses the context-dependent immune effects of SASP in tumor immune evasion and therapy resistance, and explores emerging strategies that target SnCs or remodel the immune microenvironment.

## 2 Aging-driven tumor immune escape

### 2.1 Immunosenescence

**Figure 3:**
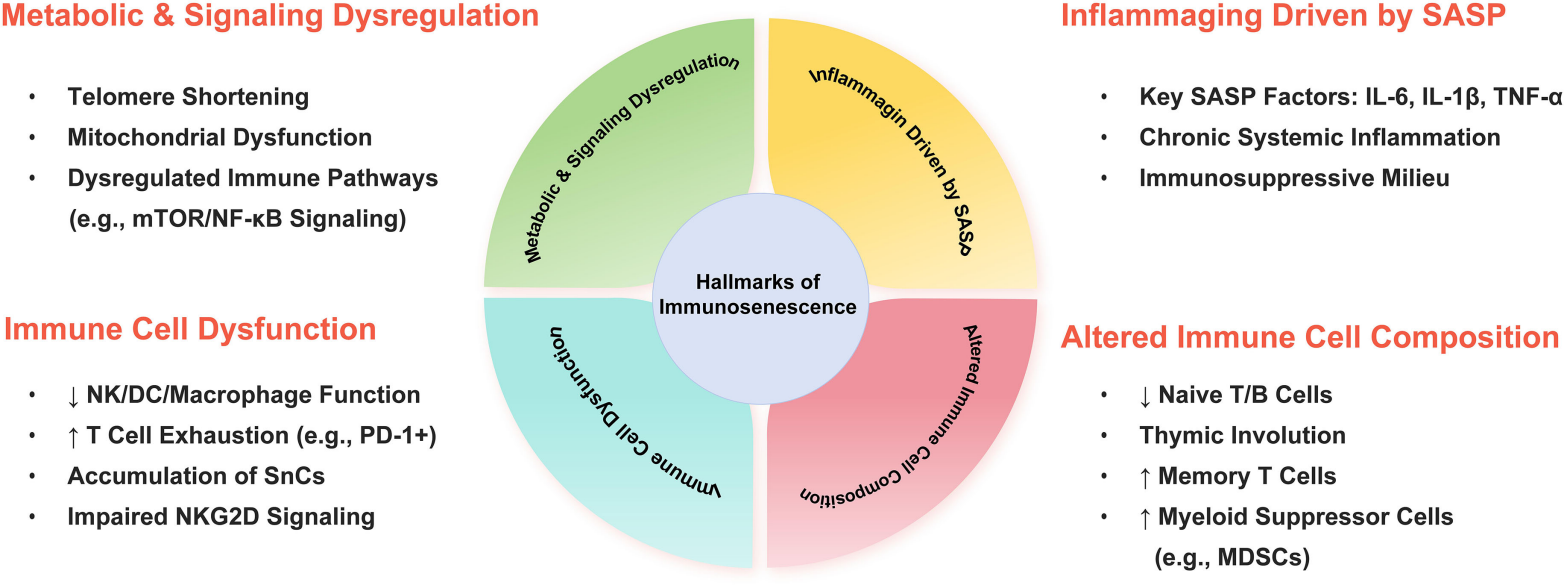
Hallmarks of immunosenescence and their pro-tumorigenic mechanisms. Immunosenescence encompasses altered immune cell composition, cellular dysfunction, SASP-driven chronic inflammation, and metabolic/signaling dysregulation. These alterations collectively compromise immune surveillance and enhance immunosuppression, notably through the accumulation of SnCs and upregulation of exhaustion markers (e.g., PD-1), thereby fueling cancer predisposition and therapy resistance. SASP, senescence-associated secretory phenotype; SnCs, senescent cells; PD-1, programmed death 1.

The concept of immunosenescence was first introduced by Roy Walford in 1964 (42). It describes the gradual decline in immune function that occurs with age, affecting both innate immunity and adaptive immunity (43). Advances in research technologies have transformed the understanding of immunosenescence from initial phenomenological observations to a systematic comprehension of multidimensional alterations at cellular, tissue, and systemic levels. Its core characteristics include structural degeneration of immune organs, loss of lymphocyte repertoire diversity, impaired effector functions, and persistent chronic inflammatory responses (44) (**Figure 3**).

In recent years, a growing body of research has shown that the aging of the immune system not only impairs immune surveillance but also plays a significant role in systemic aging. By driving organism-wide aging, immunosenescence further exacerbates the development of various age-related diseases, including cancer (45). Recent studies have revealed that the aged immune system itself can promote the accumulation of SnCs and aggravate systemic inflammation, thereby accelerating the aging process. This systemic effect of immunosenescence is particularly pronounced in older individuals and is closely linked to the onset of aging-associated diseases such as cardiovascular disorders (46), neurodegeneration (47), metabolic diseases (48, 49), and cancer (43). Specifically, through functional alterations in immune cells, immunosenescence compromises the immune system’s ability to monitor and eliminate tumors, thereby facilitating tumor initiation and progression.

Within the adaptive immune system, aging induces significant structural degeneration of immune organs, with thymic involution recognized as one of the most critical and earliest hallmark events of immunosenescence (50). As the primary site for T cell development and maturation, the thymus exhibits a strong negative correlation between its function and age. In 1998, Douek et al. pioneered the use of T cell receptor rearrangement excision circles (TRECs) as a quantitative measure of thymic output of naive T cells, demonstrating progressive age-associated structural degeneration of the thymus. This degeneration is characterized by substantial parenchymal cell loss, adipose tissue infiltration, and blurring of corticomedullary boundaries, ultimately leading to a marked decline in naive T cell production (51, 52). The insufficient output of naive T cells compromises the renewal of the peripheral T cell repertoire, forcing the organism to rely on peripheral expansion of existing memory T cells to maintain numerical homeostasis. This compensatory mechanism results in a structural imbalance within the adaptive immune repertoire (53). Furthermore, in older individuals, virus-specific T-cell clones targeting latent viruses such as cytomegalovirus (CMV) undergo clonal expansion, a phenomenon often referred to as “memory inflation.” This expansion excessively occupies immunological space, further constraining the diversity of the T-cell receptor repertoire and significantly limiting the body’s capacity to respond to neoantigens (54, 55).

The aging of the hematopoietic system plays a decisive role in immunosenescence. Although the number of hematopoietic stem cells (HSCs) may increase with age, their functional capacity declines markedly. This results in lower regenerative activity, a skewed differentiation potential favoring the myeloid lineage, and a diminished ability to respond to external signals (56–58). Consequently, this myeloid bias leads to increased production of macrophages and neutrophils, whereas the capacity for lymphopoiesis declines, making it challenging to maintain a functionally intact and diverse repertoire of B and T cells (57, 59, 60). Furthermore, senescence within the bone marrow microenvironment disrupts hematopoietic homeostasis. The functional decline of aged stromal cells compromises HSCs maintenance and differentiation, promotes immune dysfunction and inflammation (61, 62), and can enhance immune evasion by tumor cells. Additionally, structural disorganization in secondary lymphoid organs, such as lymph nodes and the spleen, further impairs the normal migration, homing, and activation efficiency of immune cells (63–65).

At the cellular level, age-related decline in immune cells is a key manifestation of immunosenescence. In aged organisms, the number of naive T cells is substantially reduced, while the proportions of highly differentiated terminal effector T cells and exhausted T cells are significantly increased (66). These exhausted T cells typically express multiple inhibitory receptors such as programmed death 1 (PD-1), T cell immunoglobulin and mucin domain-containing protein 3 (TIM-3), and lymphocyte activation gene 3 (LAG-3), and exhibit diminished cytotoxic and proliferative capacities (67). In recent years, the senescent state of human cytotoxic CD8^+^ T cells has gradually been recognized as an important component of immunosenescence and is distinguished from conventional functional exhaustion. Senescence-associated CD8^+^ T cells are generally characterized by stable cell cycle arrest, telomere shortening, accumulation of DNA damage, and a terminally differentiated phenotype marked by loss of CD28 and high expression of CD57 and killer cell lectin-like receptor subfamily G member 1 (KLRG1) (68, 69). Functionally, these cells show significantly restricted proliferative ability and antigen-specific killing, yet they can persistently secrete pro-inflammatory cytokines such as interferon-gamma (IFN-γ) and tumor necrosis factor alpha (TNF-α) (69). Recent studies indicate that senescent CD8^+^ T cells are not only a consequence of immune decline; their accumulation in aged organisms can also drive systemic aging in a feed-forward manner by amplifying chronic inflammation and promoting the formation of SnCs (69–71).

In addition to T cells, the key functions of B cells in aged individuals also undergo significant impairment (72), including decreased antibody affinity, reduced somatic mutations, and impaired class-switching responses, making it difficult for the elderly body to produce high-quality antibodies (73). This not only weakens the fight against infections but also reduces the production of effective antitumor antibodies. More importantly, senescent B cells usually remain in an inflammatory state, secreting inflammatory substances, which causes the chronic inflammatory condition to persist. At the same time, the innate immune system also declines with age. NK cells become much weaker at killing cancerous or infected cells (74). Similarly, macrophages are less able to engulf pathogens and to alert the rest of the immune system to threats (75).

The molecular basis underlying these changes includes a persistent low-level chronic inflammatory state in the body (inflammaging) (76), the continuous shortening of telomeres that limits the proliferative potential of cells (77), and the continuous accumulation of DNA damage with weakened repair capabilities (10). Ultimately, these systematic changes led to a comprehensive weakening of the core functions of the immune system, a significant decline in the ability to resist new pathogens and the monitoring function for eliminating abnormal cells (such as tumor cells), an imbalance in the ability to maintain the homeostasis of the internal environment, and a reduced response rate to vaccines. Together, all these changes lay the groundwork for subsequent SASP-mediated immunosuppression and tumor immune escape.

### 2.2 SASP

Studies have confirmed that SnCs persistently release a complex network of secretory factors, comprising various cytokines, chemokines, growth factors, and proteases, collectively known as SASP. The SASP is not only a hallmark feature of SnCs but also serves as a critical link between cellular senescence and organismal physiological and pathological changes (78–80). Although the composition of the SASP is heterogeneous (81, 82), many classic molecules, such as interleukin-6 (IL-6), interleukin-8 (IL-8), TNF-α, granulocyte-macrophage colony-stimulating factor (GM-CSF), matrix metalloproteinase-1 (MMP-1), matrix metalloproteinase-9 (MMP-9), C-C motif ligand 2 (CCL2), and vascular endothelial growth factor (VEGF), can be detected in most SnCs (81, 83).

New evidence suggests that SnCs are not in a state of immune silence. On the contrary, they are clearly immunogenic. In the early stage after the induction of senescence, the SASP recruits and activates NK cells, macrophages, and effector T cells through the secretion of inflammatory factors and chemokines and the up-regulation of stress-induced ligands, thereby promoting the immune-mediated clearance process (84–86). Notably, recent studies have shown that SnCs are able to efficiently activate dendritic cells and antigen-specific CD8^+^ T cells, in part by enhancing major histocompatibility complex class I (MHC-I) antigen presentation as well as displaying aging-related autopeptides (84). This immunogenic function helps to maintain the “equilibrium” phase of tumor immune editing, thereby inhibiting tumor initiation and early progression (87–89).

However, as SnCs persistently accumulate with advancing age or under pathological conditions, the chronically present SASP signals gradually shift from being immunologically activating to immunosuppressive. The persistent presence of numerous pro-inflammatory factors continuously activates signaling pathways such as nuclear factor kappaB (NF-κB) and signal transducer and activator of transcription 3 (STAT3), thereby enhancing the survival, proliferation, and migratory potential of tumor cells. Notably, IL-6−mediated STAT3 activation has been shown to promote the upregulation of programmed death ligand-1 (PD-L1) expression on tumor cells, which in turn suppresses CD8^+^ T cell activity and renders tumors more resistant to immune clearance (90–92). Particularly in aging individuals, the sustained activation of the IL-6/STAT3 signaling axis stems primarily from the persistent presence of SnCs and their sustained SASP release, rather than from transient acute inflammatory responses. In the elderly, this SASP-driven chronic inflammation is considered one of the key reasons for the diminished efficacy of ICIs (92).

Furthermore, SASP significantly affects the recruitment and differentiation of immune cells, promoting the accumulation of immunosuppressive cells in the tumor microenvironment. Studies have shown that SnCs recruit bone marrow-derived suppressive cells (MDSCs), tumor-associated macrophages (TAMs), and regulatory T cells (Tregs) through the secretion of chemokines such as CCL2 and chemokine (C-X-C motif) ligand 1 (CXCL1) (93–95). These cells effectively inhibit the function of effector T cells and NK cells and induce their exhaustion by releasing immunosuppressive factors such as inducible nitric oxide synthase (iNOS), arginase I (ARG1), interleukin-10 (IL-10) and transforming growth factor-beta (TGF-β), thus providing a strong immune barrier for tumors (96–98). In the context of aging, this process is often further amplified. Age-related myeloid bias of the hematopoietic system and diminished immune clearance capacity render the organism more susceptible to SASP-mediated recruitment and expansion of immunosuppressive myeloid cells. This leads to the sustained accumulation and prolonged retention of cells such as MDSCs within the tumor microenvironment.

Simultaneously, matrix metalloproteinases (MMPs) in the SASP actively break down and remodel the extracellular matrix (ECM) (99), causing its structural disorder and triggering abnormal fibrotic repair (100). Unlike transient matrix reorganization, this process represents a cumulative, age-related tissue sclerosis that physically impedes the infiltration of immune cells into the tumor core. MMPs can also cleave various members of the C-C motif chemokine ligand (CC) and C-X-C motif chemokine ligand (CXC) chemokine families, rendering them unable to attract immune cells, thereby synergistically suppressing antitumor immune responses (99). For example, when Chemokine (C-C motif) ligand 8 (CCL8) is cleaved by these proteases, it loses its antitumor function in melanoma (101). MMPs can also directly degrade NK receptor group 2 member D (NKG2D) ligands, such as major histocompatibility complex class I polypeptide-related sequence A and B (MICA/B), thereby impairing NK cell-mediated clearance of tumor cells (102). In addition, MMPs can also cleave interleukin-2 receptor-α (IL-2Rα) on T cell, thereby inhibiting the proliferation of T cell and further weakening the clearance capacity of the innate immune system (103). In summary, the multiple effects of MMPs together impair the immune response to tumor cells in aged individuals.

In summary, the SASP exhibits a distinct dual role in the occurrence and development of tumors. In the early stages, its immunogenic properties help activate immune surveillance and restrict tumor growth. However, under conditions of persistent SnCs accumulation, long-term SASP signaling drives the tumor immunoediting process from the “equilibrium” phase toward “escape.” This is achieved by amplifying inflammation, recruiting immunosuppressive cells, remodeling the extracellular matrix, and disrupting immune recognition pathways. This process is particularly pronounced in aged organisms, providing a strong rationale for intervention strategies targeting the SASP or SnCs themselves, such as senolytics and senomorphics.

### 2.3 Immune evasion by SnCs

Although SnCs can exhibit immunogenicity through the SASP in the early stages, they can evolve a series of active and sophisticated immune evasion mechanisms to resist clearance when they persistently accumulate in tissues and face sustained immune pressure.

The core of this evasion mechanism lies in the ability of SnCs to actively disrupt key immune recognition signals. The innate immune system, particularly NK cells, identifies and eliminates abnormal cells through specific receptor-ligand interactions (104–106). In aging individuals, the impairment of the NKG2D–MICA/B axis exhibits a distinct age-dependent pattern. This disruption results not only from the excessive shedding of MICA/B ligands from SnCs but also from the functional decline of NK cells themselves, thereby creating a dual barrier to immune surveillance (107, 108). NK cells and CD8^+^ T cells recognize the stress ligands MICA and MICB expressed on SnCs through their surface NKG2D receptors, thereby initiating the clearance program (109, 110). However, MMPs and a disintegrin and metalloproteinase domain-containing protein (ADAM) 10/17, which are highly expressed in SnCs, cleave the extracellular domains of these ligands through proteolysis, causing them to be shed from the membrane surface to form soluble MICA/B (sMICA/B). Binding of these soluble ligands to NKG2D receptors on immune cells induces degradation of the receptors, leading to a marked impairment of the recognition of SnCs by NK and T cells and, ultimately, to a substantial reduction in the efficacy of immune surveillance (111–113).

At the same time, SnCs also evade immune surveillance by down-regulating antigen presentation related molecules and up-regulating immunosuppressive signals. Downregulation of MHC-I molecules directly impairs antigen presentation to CD8^+^ T cells, while the expression of inhibitory ligands such as PD-L1, human leukocyte antigen-E (HLA-E), and CD47 is broadly upregulated (104, 114). Among them, PD-L1 inhibits its activation by binding to PD-1 on the surface of T cells (115). HLA-E binds to the NKG2A receptor on the surface of NK cells to transmit inhibitory signals (104). CD47 blocks macrophage phagocytosis by signaling “don’t eat me” (116). These changes collectively convert SnCs into an “immunotolerant” type of cells, enabling them to evade clearance by both innate and adaptive immune systems. SASP further aggravated this immune escape effect. Factors such as IL-6 and TGF-β in SASP components inhibit the expression of ligands in neighboring cells through paracrine effects, while inducing PD-L1 upregulation in surrounding tissues, thereby expanding the microenvironment of immunosuppression (117).

In the regulation of innate immunity, exosomes released by SnCs carry a variety of immune regulatory micrornas (such as miR-21 and miR-146a), which are able to inhibit the activation of Toll-like receptor (TLR) signaling pathway in macrophages and dendritic cells, leading to a decrease in their ability to present antigens and to inflammatory responses (118, 119). Additionally, altered glycosylation patterns on the surface of SnCs enhance their recognition by Siglec family receptors, which recognize SnCs as “self” components, further reducing immune system attack (120, 121).

In summary, SnCs establish effective immune escape mechanisms by regulating immune ligand-receptor interactions at multiple levels, including weakening activation signals, enhancing inhibitory signals, reducing the efficiency of antigen presentation, and altering the recognition characteristics of innate immunity. These mechanisms not only promote the continuous accumulation of SnCs in tissues, but also provide immune escape pathways for tumor cells. Therefore, targeted intervention strategies, such as inhibiting ADAM10/17 activity and restoring NKG2D signaling axis function, may become an important research direction for enhancing antitumor immune responses in elderly individuals.

### 2.4 Dysregulated immune signaling pathways

Aging affects not only ligand-receptor interactions on the cell surface but also disrupts key signaling pathways that maintain immune homeostasis and stress responses at multiple levels. These changes collectively establish a tissue microenvironment in aged individuals that favors immune evasion (122). In terms of antigen presentation, SnCs often have decreased expression of MHC-I molecules, reduced immunoproteasome activity, and weakened transporter Associated with Antigen Processing 1 and 2 (TAP1/2) transport function, resulting in the inability to be efficiently processed and presented to CD8^+^ T cells for tumor-associated antigens and neoantigens (123). As a result, the adaptive immune system is less capable of recognizing abnormal cells, and the antigenic foundation necessary for immune checkpoint inhibitor efficacy is also undermined.

Dysregulated expression of immune checkpoint molecules represents another hallmark of the aging microenvironment. Inhibitory molecules such as PD-L1, TIM-3, and Galectin-9 are significantly upregulated in SnCs and under chronic inflammatory conditions (114, 124). This upregulation is closely associated with the persistent activation of NF-κB and STAT3 signaling pathways (125). By suppressing the proliferation and cytotoxic function of effector T cells, these molecules promote T cell exhaustion, creating a state in which the immune system can recognize target cells but fails to effectively eliminate them (124).

The impairment of key tumor-suppressor pathways also plays a central role in immune evasion during aging. Notably, the functional decline of mutations in the tumor suppressor p53 (p53) and p16INK4a signaling is frequently observed. Under physiological conditions, p53 induces cell cycle arrest, apoptosis, and DNA repair, thereby preventing the proliferation of abnormal cells (126). However, under prolonged stress, SnCs often exhibit attenuated p53 signaling, which enhances their tolerance to DNA damage and increases the risk of mutation accumulation (127, 128). Meanwhile, sustained high expression of p16INK4a drives cellular senescence but also disrupts cell cycle regulation and indirectly affects genes involved in antigen processing and immune activation, thereby weakening immune surveillance against potential tumor cells (129, 130).

Furthermore, evolutionarily conserved pathways such as Wnt, Notch, and TGF-β form a synergistic regulatory network during aging that promotes immune evasion. Persistent TGF-β signaling inhibits NK cell function, promotes Treg differentiation, and downregulates NKG2D ligand expression (131–133). Aberrant activation of the Wnt/β-catenin pathway is closely associated with impaired dendritic cell recruitment and reduced T cell infiltration into tumors (134). Together, these pathways shape a tumor microenvironment with strong immunosuppressive properties, effectively creating a “safe haven” for tumor growth within aging tissues.

In summary, age-related dysfunction of key proteins and dysregulation of signaling pathways not only weaken intrinsic tumor-suppressor mechanisms but also profoundly impair the immune system’s capacity to recognize and eliminate target cells. Together, these alterations create favorable conditions for tumor cells to evade immune surveillance in the elderly.

## 3 Immunotherapeutic strategies

With the accelerating aging of the global population, elderly individuals now represent a growing proportion of cancer patients. However, due to factors such as immunosenescence, chronic inflammation, and a higher burden of comorbidities, older patients generally show poorer responses to current immunotherapies compared to younger individuals (135). Consequently, optimizing immunotherapeutic strategies in the context of aging has become a major focus in oncology research. In recent years, multiple approaches have been explored, including the elimination of SnCs (136), modulation of SASP (137), enhancement of immune function (138), and control of chronic inflammation (139), with the goal of improving treatment outcomes and survival in the elderly.

### 3.1 Senolytic therapy

In the context of aging, the persistent accumulation of SnCs is considered a key driver of both immune function decline and the heightened immunosuppressive nature of the tumor microenvironment. Consequently, directly eliminating SnCs, known as senolytic therapy, has emerged as a core strategy for improving the immune status in elderly cancer patients (136). SnCs achieve prolonged survival by activating various senescence-associated anti-apoptotic pathways (SAAPs). Senolytics function by precisely targeting these pathways, selectively inducing apoptosis in SnCs and thereby alleviating the aging-related immunosuppressive milieu at its source.

Reported senolytics include Dasatinib (140), Quercetin (141), and Bcl-2 family inhibitors such as Navitoclax (142). These agents interfere with the anti-apoptotic signals that SnCs rely on, rendering them more susceptible to cell death. Multiple animal studies have demonstrated that clearing SnCs can enhance T-cell proliferation and effector function, improve the efficacy of ICIs such as anti-PD-1 therapy, and to some extent delay tumor initiation and progression (143, 144).

In the setting of cancer therapy for the elderly, senolytics are more likely to be used in combination with immunotherapy rather than as monotherapy. On one hand, short-term or intermittent clearance of SnCs may help reshape the immune microenvironment and lower the threshold of immunosuppression, thereby increasing the likelihood of a response to subsequent immunotherapy (145). On the other hand, SnCs still contribute to tissue integrity and repair processes in certain contexts, and their broad elimination could lead to tissue dysfunction and systemic toxicity. Therefore, defining the appropriate patient population, optimal dosing schedule, and therapeutic window for senolytics represents a critical challenge in their clinical translation (146).

### 3.2 SASP-targeted therapy

In contrast to senolytics, which directly eliminate SnCs, strategies aimed at modulating the SASP, known as senomorphics, do not seek to remove SnCs. Instead, they work by suppressing the persistent release of inflammatory and immune-disruptive signals, thereby ameliorating the tissue and immune microenvironment in the context of aging (137).

**Figure 4:**
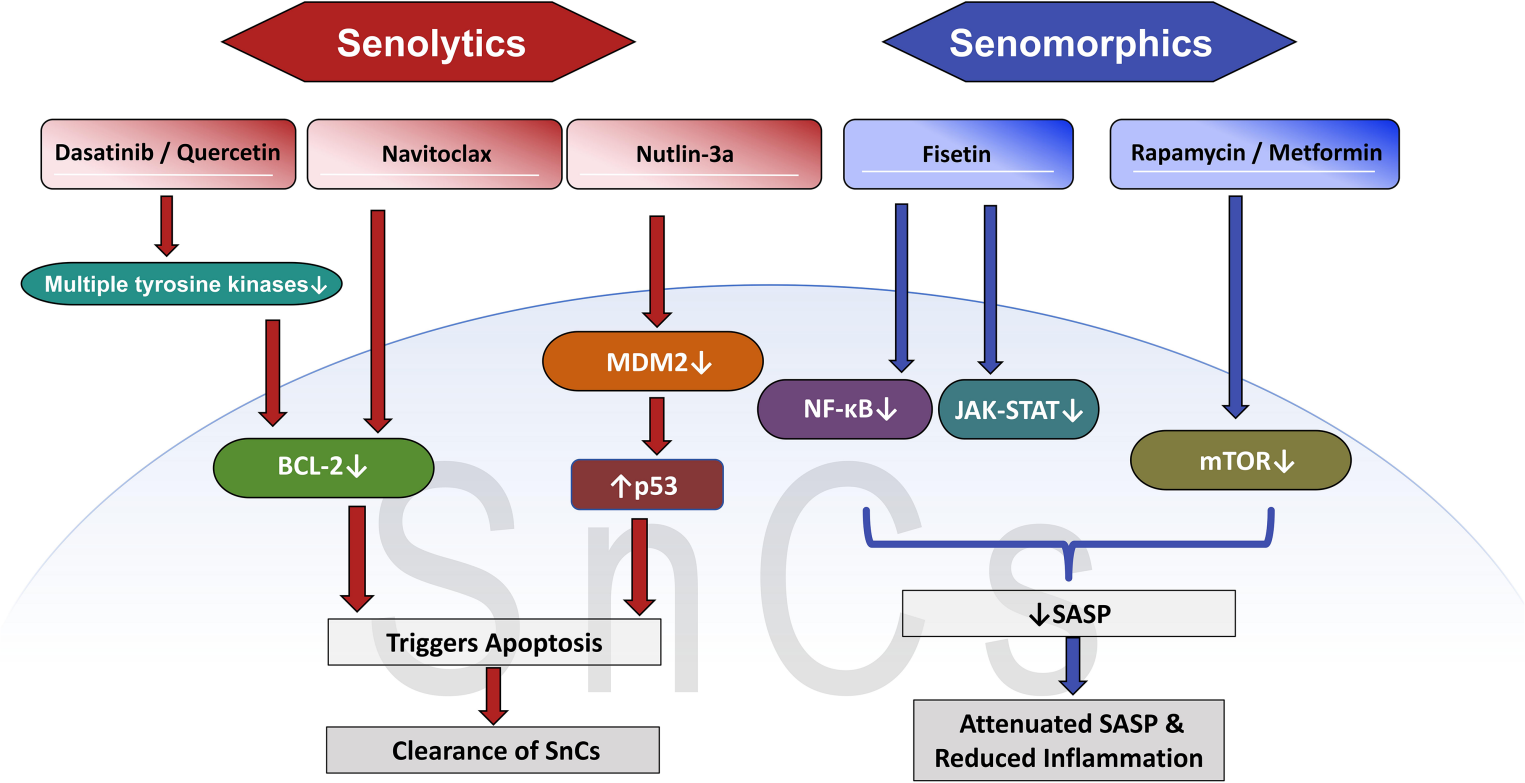
Mechanisms of action for senolytic and senomorphic therapies. The diagram compares two strategies: senolytics (left) that directly eliminate SnCs by inducing apoptosis via targets like BCL-2 or p53, and senomorphics (right) that suppress the harmful SASP by inhibiting signaling pathways such as mTOR or NF-κB. SnCs, senescent cells; BCL-2, B-cell lymphocyte/leukemia 2 gene; p53, mutations in the tumor suppressor p53; SASP, senescence-associated secretory phenotype; mTOR, mammalian target of rapamycin; NF-κB, nuclear factor kappaB.

Promising senomorphic agents under investigation include metformin (147), rapamycin (148), and JAK inhibitors (**Figure 4**). Rapamycin, by inhibiting the mTOR signaling pathway, can reduce SASP production in SnCs and alleviate the aging-related chronic low-grade inflammatory state known as inflammaging (149). JAK inhibitors, on the other hand, directly interfere with the downstream signaling of key inflammatory cytokines such as IL-6 and IL-8, thereby attenuating the persistent disruptive influence of SnCs on the immune microenvironment without affecting cell viability (150).

In the context of cancer therapy, the potential value of SASP modulation lies more in its “environmental optimization” effect rather than in direct anti-tumor activity. By mitigating chronic inflammation and aging-related immune imbalance, senomorphics may help lower the threshold of immunosuppression, creating more favorable conditions for responses to ICIs or cellular therapies. This approach could be particularly relevant for elderly patient populations with pronounced immunosenescence.

However, it is crucial to emphasize that the SASP exhibits significant context-dependency and functional duality. It still plays physiological roles in processes such as acute stress responses, tissue repair, and damage clearance. Consequently, long-term or excessive suppression of the SASP could adversely affect tissue regeneration, immune homeostasis, and even anti-infective capacity (145, 151). Striking a balance between inhibiting chronic inflammation and preserving physiological function, along with determining appropriate dosing and timing, represents a key challenge for the safe clinical translation of SASP modulation strategies in elderly cancer patients.

### 3.3 Immune restoration

Given that immunosenescence is a key determinant of heterogeneous responses to immunotherapy in elderly patients, directly restoring or enhancing the function of the aged immune system is considered a crucial therapeutic strategy. In contrast to younger patients, who primarily benefit from immune activation, interventions in the elderly should focus more on repairing structural defects and functional declines within the immune system. This approach aims to restore its foundational capacity for tumor surveillance. Such strategies include vaccines, cell-based therapies, ICIs, and lifestyle interventions (152–154). At the level of adaptive immunity, vaccine strategies have regained attention in recent years for the elderly. By enhancing antigen presentation efficiency and promoting naive T cell activation, vaccination can partially compensate for the diminished naive T cell reservoir caused by immunosenescence, thereby boosting subsequent immune responses (152).

Meanwhile, the application of cell-based therapies such as chimeric antigen receptor (CAR) T-cell therapy in elderly patients, though limited by hematopoietic aging and reduced T cell quality, has shown potential. Studies indicate that functional status and therapeutic potential of T cells derived from older individuals can be improved to some extent through metabolic or signaling pathway interventions, such as pretreatment with metformin or rapamycin (155).

Furthermore, ICIs have achieved significant progress as a major treatment modality for elderly patients with cancer (156). Although immunosenescence and chronic inflammation may attenuate responses to ICIs in some, both clinical trials and real-world data suggest that some elderly patients can derive survival benefits comparable to those of younger patients (157). However, immunosenescence is not a uniform process, and significant inter-individual variation in immune function exists. There is an urgent need for patient stratification based on immunobiological features rather than chronological age alone.

Additionally, lifestyle interventions, such as regular exercise, optimized nutrition, and sleep management, represent low-risk, sustainable approaches. These have been shown to improve immune homeostasis, reduce chronic inflammation, and to some extent enhance the tolerability and durability of immunotherapy (45). In elderly patients, such interventions can serve as valuable adjuncts to pharmacotherapy, providing foundational support for immune functional restoration.

### 3.4 Optimizing immune checkpoint therapy for the elderly

ICIs have become a cornerstone in the treatment of various cancers. However, the therapeutic response to ICIs in elderly patients exhibits significant heterogeneity in clinical practice (158, 159). While immunosenescence and a chronic inflammatory state are thought to potentially attenuate ICI efficacy, previous clinical studies and real-world data indicate that a subset of elderly patients can still achieve survival benefits comparable to those of younger patients (160). The efficacy is influenced by a combination of factors including tumor type, degree of immunosenescence, comorbidities, and prior treatment history, suggesting that chronological age alone is insufficient as a sole predictor for ICI response (161, 162). More refined immunobiological stratification and individualized assessment are particularly crucial for making immunotherapy decisions in this population.

In this context, anti-inflammatory interventions have been proposed as a potential strategy to optimize ICI responses in the elderly. Inflammatory cytokines such as IL-6 and TNF-α are chronically elevated in older individuals, contributing to tumor immune escape. Therefore, anti-inflammatory therapies, including IL-6 antibodies, TNF-α blockers, and NSAIDs, may improve the tumor microenvironment and enhance immune clearance (163). Some studies suggest that IL-6 blockade can augment the anti-tumor effects of ICIs (164). However, other clinical research, such as in pancreatic cancer patients where combining ICIs with radiotherapy showed efficacy, found that adding an IL-6 receptor antibody conferred no additional benefit (165). Thus, the application of anti-inflammatory therapy in elderly cancer immunotherapy requires more precise patient stratification and prospective validation.

In summary, the field of immunotherapy is evolving from a focus on “directly activating the immune system” towards “improving the aged microenvironment.” Strategies encompassing SnCs clearance, SASP modulation, immune function enhancement, and chronic inflammation suppression collectively offer new possibilities for improving immunotherapy outcomes in elderly cancer patients.

## 4 Conclusions and future perspectives

Aging profoundly affects the tumor immune microenvironment through multiple biological pathways. This effect is not only reflected in the degradation of the structure and function of the immune system, but also in the continuous remodeling of the local microenvironment by SnCs and SASP secreted by them. Immunosenescence can lead to decreased antigen presentation efficiency, decreased diversity of T cell repertoire and weakened innate immune function, which significantly impairs the immune surveillance ability of the aged body. At the same time, SnCs further promote the immune escape of tumor cells by changing the immune ligand-receptor signaling, activating the immunosuppressive pathway, and promoting the accumulation of immunosuppressive cells. These changes together constitute an important biological basis for the increased incidence of cancer, faster disease progression, and different responses to immunotherapy in the elderly population.

In the future, with the deepening of immunotherapy in tumor treatment, how to improve the response of immunotherapy under the background of aging has become a key issue to be solved urgently. Senolytics and senomorphics have brought new directions for improving the aging microenvironment. Restoring the function of immune cells through metabolic and epigenetic regulation is expected to enhance the immune efficacy from the root. Strategies targeting immunosuppressive cells such as MDSCs, TAMs and Tregs are expected to further reshape the tumor immune ecology. In addition, the combination of multiple immune checkpoint blockade, immunotherapy and anti-inflammatory therapy, and a more accurate and individualized immune evaluation system may break through the bottleneck of immune tolerance and insufficient efficacy in elderly patients.

In summary, a deeper mechanistic understanding of how aging promotes immune escape will provide a critical foundation for developing next-generation immunotherapies tailored to older patients. Future research needs to integrate aging biology, tumor immunology and precision medicine to provide more effective and sustainable solutions for cancer prevention and treatment in an aging society.
